# Plasma Hepatic Transaminases and Incidence of Metabolic Syndrome Before Midlife in Military Adults: A CHIEF Cohort Study

**DOI:** 10.2174/0118715303326392241022050205

**Published:** 2025-01-09

**Authors:** Fang-Chen Liu, Kai-Wen Chen, Kun-Zhe Tsai, Chen-Chih Chu, Yen-Chen Lin, Yun-Chen Chang, Gen-Min Lin

**Affiliations:** 1 Department of Medicine, Hualien Armed Forces General Hospital, Hualien City, Taiwan;; 2 Department of Medicine, Tri-Service General Hospital, National Defense Medical Center, Taipei, Taiwan;; 3 Department of Stomatology of Periodontology, Mackay Memorial Hospital, Taipei, Taiwan;; 4 Department of Medicine, Linkou Chang Gung Memorial Hospital, Taoyuan, Taiwan;; 5 School of Nursing and Graduate Institute of Nursing, China Medical University, Taichung, Taiwan;; 6 Department of Nursing, China Medical University Hospital, Taichung, Taiwan

**Keywords:** Alanine transaminase, aspartate transaminase, metabolic syndrome, military personnel, nonalcoholic fatty liver disease, young adults

## Abstract

**Background:**

Plasma AST and ALT may reflect the nonalcoholic fatty liver disease (NAFLD) severity and have been associated with the risk of MetS in middle- or old-aged individuals.

**Aims:**

This study aimed to examine the associations of plasma hepatic aspartate and alanine transaminases (AST and ALT) levels with incident metabolic syndrome (MetS) in young adults, which have not been verified before.

**Objective:**

The goal of this study was to identify the association between plasma hepatic transaminases and the incidence of new-onset MetS among young adults.

**Methods:**

There were 2,804 military men and women, with ages of 18-39 years, free of baseline MetS and any viral hepatitis in Taiwan in 2014. Incident MetS were followed in the annual military health examinations from baseline till the end of 2020. The definition of MetS was made using the criteria of the International Diabetes Federation. Plasma concentrations of AST and ALT were checked at baseline. A Multivariable Cox regression model with adjustments for sex, age, each component of MetS, body mass index, substance use status, and physical activity at baseline was performed to determine the associations. Subgroup analyses were performed according to sex and each MetS component.

**Results:**

During a median follow-up of 5.8 years, 644 incident MetS (23.0%) developed. ALT and AST levels (each 10 U/L increase) were respectively associated with 5% and 11% increased risk of incidence of MetS (hazard ratios (HRs) and 95% confidence intervals (CIs): 1.05 (1.01-1.09) and 1.11 (1.04-1.19), respectively). In subgroup analyses, the risk of incidence of MetS with ALT and AST levels respectively increased 75% and 114% in women (HRs: 1.75 (1.07-2.87) and 2.14 (1.35-3.41), respectively), and 7% and 14% in those free of central obesity (HRs: 1.07 (1.02-1.11) and 1.14 (1.06-1.23), respectively) which were higher than their counterparts (*p*-values for interaction by sex: 0.06 and 0.001, respectively; and by central obesity: 0.04 and 0.07, respectively).

**Conclusion:**

Plasma hepatic transaminase levels were positively associated with incident MetS among young adults. The individual role of central obesity and sex on the association of ALT and AST with incident MetS should be further clarified.

## 1 INTRODUCTION

Metabolic syndrome (MetS) is a cluster of disorders that significantly impacts public health. Based on the guideline established by the International Diabetes Federation (IDF), MetS is characterized by some features, *i.e.,* dyslipidemia (elevated triglycerides and reduced high-density lipoprotein cholesterol (HDL-C), hyperglycemia (insulin resistance), hypertension, and central obesity [1, 2]. People with MetS face an elevated risk of developing type 2 diabetes mellitus and cardiovascular disease [3-7]. A concerning trend of increasing incidence of MetS is also observed worldwide. The prevalence of MetS varies across regions, with rates of 34.7% in the U.S., 21.1% to 28.9% in Asia, and 24.3% in Europe [8-12].

Non-alcoholic fatty liver disease (NAFLD) represents a condition characterized by fat accumulation in more than 5% of hepatocytes in the absence of excessive alcohol consumption or other chronic liver disease, *e.g.,* viral hepatitis [13, 14]. Aside from chronic hepatitis C [15], NAFLD stands as one of the leading causes of liver transplantation worldwide. Non-alcoholic steatohepatitis (NASH) was observed in 10-20% of the NAFLD cases [16], accompanied by elevated plasma hepatic transaminase levels [17, 18], and 20-50% of the NASH cases might lead to liver cirrhosis within 10 years [19]. Though the disease has no specific clinical symptoms, it is often linked to key metabolic abnormalities, *e.g.,* dyslipidemia, obesity, and insulin resistance -three hallmark features of MetS [20-23]. In 2023, the term metabolic dysfunction-associated steatotic liver disease (MASLD) [24], defined as the presence of excess triglyceride storage in the liver in the presence of at least one cardiometabolic risk factor, was officially used to replace NAFLD, particularly among lean individuals. Some studies have revealed an association between hepatic transaminases and MetS [25-30], whereas the risk of new-onset MetS before midlife and the modifiers, such as sex and obesity in the association, has not been explored. Therefore, this study aimed to examine the association between plasma hepatic transaminases and incident MetS in young adults.

## 2 METHODS

### 2.1 Study Population

The cohort study population comprised 4,080 military men and women aged between 18 and 50 years who had no baseline diabetes and did not take any medications in 2014. All the data were collected from the “Cardiorespiratory Fitness and Health in Eastern Armed Forces” (CHIEF) study in Taiwan [31-34]. From Jan 1, 2014, to Dec 31, 2020, participants received comprehensive health examinations each year for assessments of the incidence of MetS. The exclusion criteria included those with baseline MetS, age ≥40 years, or chronic viral hepatitis and those who lost follow-up. This cohort study was performed in compliance with the Declaration of Helsinki principles. In addition, the study design and protocol have been approved by the Ethical Review Board of the Mennonite Christian Hospital (certificate No. 16-05-008) in Hualien, Taiwan. Further, written informed consent was obtained from all participants.

### 2.2 Annual Health Examinations (2014-2020)

The measurements of each participant's waist circumference (WC), body height, and weight were taken while they were in a standing position. To calculate the body mass index (BMI), an individual's body weight (kg) was divided by the square of their body height (m^2^). Overweight was defined as BMI of 25.0 - 29.9 kg/m^2^, and general obesity was defined as BMI ≥30.0 kg/m^2^ according to the criteria of the World Health Organization [35]. Each participant's resting blood pressure (BP) was measured once over the right arm through an automatic BP device using the oscillometric method (FT201 Parama-Tech Co., Ltd, Fukuoka, Japan). If the initial BP level was ≥130/80 mmHg, a second measurement of the BP level was taken following a 15-minute rest period. The final BP level was determined as an average of the initial and the second BP levels. The mean BP was defined as 1/3 * systolic BP + 2/3 * diastolic BP [36]. Additionally, plasma fasting glucose, total cholesterol, high-density lipoprotein cholesterol (HDL-C), triglycerides, aspartate transaminase (AST), and alanine transaminase (ALT) levels were measured using an enzymatically auto analyzer. (AU640, Olympus, Kobe, Japan) [37, 38]. The blood sample of each participant was collected after a mandatory overnight fast of 12 hours.

### 2.3 Definition of the Baseline and Incident MetS

Based on the criteria established by the IDF for the Chinese adult population [1, 2]. MetS is defined as the presence of three or more clinical features, which include: (1) serum triglycerides ≥150 mg/dL, or with lipid-lowering medications; (2) HDL-C <40 mg/dL in men and <50 mg/dL in women; (3) WC ≥90 cm in men and ≥80 cm in women; (4) fasting glucose ≥100 mg/dL, or with antidiabetic medications; (5) systolic BP ≥130 mmHg, and/or diastolic BP ≥85 mmHg, or with antihypertensive medications [38-40].

### 2.4 Definition of Insulin Resistance Indices

Since hemoglobin A1c and fasting insulin levels were not available in this study, we used three noninsulin-based indices for the assessment of insulin resistance. (1) The ratio of TG/HDL-C was defined as TG divided by HDL-C [41]. (2) The triglyceride glucose (TyG) index was defined as ln (TG * fasting glucose/2) [42], and (3) the metabolic score for insulin resistance (METS-IR^)^ was defined as ln((2 * fasting glucose)+TG)* BMI/(ln(HDL-C))) [43].

### 2.5 Definition of Increased Hepatic Transaminases

According to a prior study finding by Prati *et al.,* it was noted that even within the normal range, modestly increased plasma ALT ≥30 U/L in men and ≥20 U/L in women were associated with the development of new-onset MetS [44]. In addition, we divided levels of AST, ALT, and ALT/AST ratio, which have been considered sensitive markers to insulin resistance degree [45, 46], into sex-specific quartiles separately for an assessment of the graded associations for the development of new-onset MetS.

### 2.6 Covariates of Substance Use and Physical Activity

Substance use condition, *i.e.,* tobacco smoking, betel nut chewing, and alcohol consumption, was defined as active and former/never, and moderate-intensity physical activity was assessed by weekly running time (<150 hours (hrs)/week (wk), 150-299 hrs/wk and ≥300 hrs/wk) in the past half year. The information was self-reported in a questionnaire at baseline at the Hualien Armed Forces General Hospital (2014).

### 2.7 Statistical Analysis

The baseline characteristics of the military cohort were presented as mean ± standard deviation (SD) for continuous variables and numbers (percentage) for categorical variables. The follow-up of each participant began at the baseline in 2014 and extended until the first occurrence of MetS, loss to follow-up, or the end of the follow-up period on December 31, 2020.

Kaplan-Meier Curve was used to conduct survival analysis (free of incident MetS) in each sex-specific quartile group of plasma AST, ALT, and ALT/AST ratio. Differences between the quartile groups were compared using the log-rank test. Multivariable linear regression analysis was utilized to determine the correlations between hepatic transaminases, lipid biomarkers, fasting glucose, and insulin resistance indices with simultaneous adjustments for baseline age, sex, BMI, smoking status, alcohol intake status, betel nut chewing status, and physical activity. Multivariable Cox proportional hazards regression analysis was used to determine the associations of AST, ALT, and ALT/AST ratio, which were separately treated as continuous variables and categorical variables by sex-specific quartiles with incident MetS. The covariates in the multivariable Model 1 were the same as the adjusted covariates in linear regression analysis. Triglycerides, fasting glucose, WC, mean BP, and HDL-C were further adjusted in Model 2. These covariates associated with MetS in previous studies were selected in the multivariable Models. In addition, the risk of incident MetS with increased ALT levels defined by Prati *et al.* (≥30 U/L in men and ≥20 U/L in women) [41], Suh *et al.* for Koreans (≥27 U/L in men and ≥15 U/L in women) [44] and this study findings using receiver operating characteristic (ROC) was determined with multivariable adjustments in Model 2. Statistical power was estimated according to each group size and incidence of MetS with Type 1 error 0.01 [45]. We conducted stratified analyses to explore the associations between plasma hepatic transaminases and incident MetS within sex, BMI status (with *vs.* without obesity/overweight), BP status (with *vs.* without increased BP), fasting glucose status (with *vs.* without hyperglycemia), lipids status (with *vs.* without dyslipidemia), alcohol consumption status (active *vs.* never/former), and smoking status (active *vs.* never/former). Furthermore, formal tests for multiplicative interactions were also performed. A value of two-tailed *p* <0.05 was considered statistically significant. All statistical analyses were performed using the software SPSS v25.0 for Windows (IBM Corp., Armonk, NY, USA). (supplementary figure).

## 3 RESULTS

### 3.1 Baseline Group Characteristics

In the original cohort (N =4,080), participants with baseline MetS (N = 457), age ≥40 years (N = 58), and a history of chronic viral hepatitis B or C (N =86), and those lost to follow-up due to relocation from the military bases in Eastern Taiwan (N = 675) to the other bases were excluded. Consequently, the final cohort for analysis consisted of 2,804 subjects. The flow diagram in Fig. (**1**) reveals the selection of participants for this cohort study. During a median follow-up of 5.8 years, 644 incident MetS (22.0%) developed.

Table **1** outlines the baseline characteristics of the study cohort. There were 431 participants (15.4%) with increased ALT who developed 175 new-onset MetS, and the other 2,373 subjects with normal ALT (84.6%) who developed 469 new-onset MetS before midlife. There was a greater prevalence of men and active betel nut consumers in the increased ALT group. Notably, those with increased ALT also had a higher mean AST level and a higher ALT/AST ratio. In addition, those with increased ALT had a greater mean level of BMI and total cholesterol, and each MetS biomarker had a lower mean HDL-C level. The statistical power to the assessment of incident MetS using the definition for increased ALT levels was 100% (Table **S1**).

### 3.2 Correlations Between Hepatic Transaminases, Lipid Profile, Fasting Glucose, and Insulin Resistance Indices

Table **2** demonstrates the results of multivariable linear regression analysis for the correlations of plasma ALT, AST, and ALT/AST with lipid profiles, fasting glucose, and insulin resistance indices. For ALT, the highest Pearson correlation coefficient (r) was found with TG (r =0.421), followed by the TG/HDL-C ratio (r =0.416). The least coefficients were observed with HDL-C and fasting glucose (r =0.382). For AST, the greatest correlation coefficient was observed with TG (r =0.315), followed by total cholesterol (r =0.312), and the least coefficient was observed with METS-IR (r =0.291). For ALT/AST, the greatest coefficient was observed with the TyG index (r =0.482), followed by METS-IR (r =0.481), and the least coefficient was observed with fasting glucose (r =0.441). Fig. (**S1**) reveals the scatter plot graphs of ALT, AST, and ALT/AST against insulin resistance indices. Obviously, ALT/AST had the strongest correlation with each insulin resistance index among the plasma hepatic transaminases. In addition, the results of correlations between lipid profiles, fasting glucose, and various insulin resistance indices are shown in Table **S2**. Notably, there were high correlations (r >0.70) between TG and each insulin resistance index and between each insulin resistance index (>0.80).

### 3.3 Associations of Plasma ALT, AST, and ALT/AST with Incident Mets

**Fig. (2):**
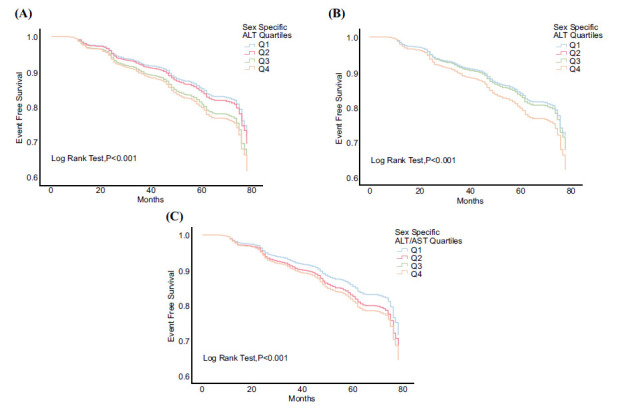
The results of the Kaplan-Meier Curve analysis for plasma ALT, AST, and ALT/AST ratio and a follow-up of new-onset metabolic syndrome (MetS) were revealed. (**A**) The highest incidence of new-onset MetS was found in the highest ALT Q4, followed by Q3, Q2, and Q1 in order (*p* <0.001). (**B**) The highest incidence of new-onset MetS was found in the highest AST Q4, followed by Q3, Q2, and Q1 in order (*p* <0.001). (**C**) The highest incidence of new-onset MetS was found in the highest ratios Q3 and Q4, followed by Q2 and Q1 in order (*p* <0.001).

Fig. (**2A**) reveals the results of the Kaplan-Meier Curve analysis for the ALT quartiles, with the highest incidence of new-onset MetS in the highest ALT Q4, followed by Q3, Q2, and Q1 in order (*p* <0.001 by log-rank test). The pattern for the ALT quartiles was consistent with that for the AST quartiles (Fig. **2B**) and the ALT/AST quartiles (Fig. **2C**) (both *p* <0.001).

Table **3** demonstrates the multivariable Cox regression analysis results for the association of plasma ALT, AST, and ALT/AST with incident MetS. As plasma ALT was treated as a continuous variable (each 10 U/L increase), ALT was associated with a 5% increased risk of incident MetS in multivariable Model 2 (hazard ratio (HR) and confidence interval: 1.05 (1.01-1.09)). As plasma ALT was classified by quartiles (Q1-Q4), there were graded associations for higher ALT quartiles with higher incidences of MetS (from Q1 (reference), +7% risk in Q2, +33% risk in Q3, to +42% risk in Q4) (HRs: 1.07 (0.79-1.45), 1.33 (1.00-1.76), 1.42 (1.07-1.88), respectively). When plasma AST was treated as a continuous variable (each 10 U/L increase), AST was associated with an 11% increased risk of incident MetS in multivariable Model 2 (HR: 1.11 (1.04-1.19)). As plasma AST was classified by quartiles (Q1-Q4), there were graded associations for higher AST quartiles with higher incidences of MetS (from Q1 (reference), +4% risk in Q2, +6% risk in Q3, to +29% risk in Q4) (HRs: 1.04 (0.79-1.37), 1.06 (0.82-1.36), 1.29 (1.01-1.65), respectively). When the ratio of ALT/AST was treated as a continuous variable (each 1 unit higher), ALT/AST was associated with a 46% increased risk of incident MetS in Model 1 (HR: 1.46 (1.21-1.77)), whereas the association was not significant in Model 2. As the ALT/AST ratio was classified by quartiles (Q1-Q4), there were graded associations for higher ALT/AST quartiles and higher incidences of MetS (from Q1 (reference), +24% risk in Q2, +59% risk in Q3, to +72% risk in Q4) (HRs: 1.24 (0.93-1.66), 1.59 (1.20-2.10), 1.72 (1.30-2.26), respectively) in Model 1. However, the graded associations with the higher ratio quartiles were blunted in Model 2.

In Table **S1**, although increased ALT levels defined by Prati *et al.* [38] were associated with a 21% increased risk of incident MetS in Model 1 (HR: 1.21 (1.01-1.45)), while the association was not significant with adjustments for baseline MetS components in Model 2. The condition was similar to increased ALT levels as defined by Suh *et al.* for Koreans [41]. When the cut-off levels for increased ALT in this study were determined by ROC analysis (≥19 U/L in men and ≥13 U/L in women) from Table **S3**, increased ALT levels were associated with 51% increased risk of incident MetS in Model 1 (HR: 1.51 (1.27-1.79)) and 28% increased risk in Model 2 (HR: 1.28 (1.08-1.53)). It was notable that all the statistical power using various cut-off levels for increased ALT to assess incident MetS was 100%.

### 3.4 Subgroup Analysis

Table **4** shows the results of subgroup analyses. The risk of incident MetS with ALT and AST levels (each 10 U/L higher) was significantly greater in women (HRs: 1.75 (1.07-2.87) and 2.14 (1.35-3.41), respectively), and in those without central obesity (HRs: 1.07 (1.02-1.11) and 1.14 (1.06-1.23), respectively), and in those without hypertriglyceridemia (HRs: 1.06 (1.02-1.11) and 1.17 (1.08-1.27), respectively) than their counterparts (*p*-values for interaction based on sex: 0.06 and 0.001, respectively; and based on the WC: 0.04 and 0.07, respectively; and based on the plasma triglycerides levels: 0.07 and 0.07, respectively). In addition, the risk of incident MetS with ALT levels was significantly greater in those without low HDL-C than in their counterparts (*p*-value for interaction: 0.06). By contrast, the risk of incident MetS with AST levels was significantly greater in those free of hypertension compared to their counterparts (*p*-value for interaction: 0.08). With regard to alcohol and cigarette consumption status, there were no statistically significant differences in the risk of new-onset MetS between active consumers and never/former consumers. It was notable that there were no differences by subgroups for the association between ALT/AST ratio and incident MetS.

## 4 DISCUSSION

This study demonstrated a significant link between levels of plasma hepatic transaminases and the risk of new-onset MetS with adjustments for potential baseline covariates, *i.e.,* age, sex, BMI, substance use, and physical activity in military young adults. The associations for ALT and AST levels persisted with additional adjustments for all components of MetS at baseline, including WC, BP, plasma triglycerides, HDL-C, and fasting glucose, while the association for ALT/AST ratio, an insulin resistance marker, was no longer significant. It was notable that the associations for ALT and AST levels were particularly greater in women without central obesity and those without dyslipidemia.

ALT is a more specific hepatic enzyme that may increase in the presence of hepatic steatosis or NAFLD [13]. Although the pathogenesis of NAFLD is not fully understood, the existing hypothesis suggests that the inflammatory pathway activated by free fatty acids, mainly produced by diet and obesity, could induce insulin resistance and the accumulation of hepatic fat. Then, the accumulation of hepatic fat triggers oxidative stress, which can result in hepatocyte injury [37-40]. Insulin resistance and obesity emerge as potential connections between NAFLD and the development of MetS. Many previous cross-sectional studies have revealed the association between ALT levels and prevalent MetS [25, 28, 29]. Notably, in this study, the association of ALT levels with incident MetS might be independent of the presence of insulin resistance and obesity among young adults, indicating that those with NAFLD with minimal liver injury at early ages were at higher risk of developing MetS in their later lives.

Compared to the commonly acceptable reference for the increased ALT levels defined as ≥30 U/L in men and ≥20 U/L in women [44] from the general population, we observed that the ALT threshold for a greater risk of new-onset MetS (≥19 U/L in men and ≥13 U/L in women) was lower among young adults. Furthermore, the increased ALT levels for a greater risk of incident MetS in the 2005 Korean National Health and Nutrition Examination Survey were defined as ≥27 U/L in men (Q4) and ≥15 U/L in women (Q3 and Q4) in their population with a mean age of 45 years [47], which were also lower than the increased ALT levels defined by the Prati *et al.* [44]. Further, our findings were close to the highest ALT quartile level in men (≥25 U/L) and women (≥14 U/L), respectively in the Korean population study [47]. Thus, the sex-specific cut-off levels of increased ALT at greater risk of MetS may vary by age and race/ethnicity, and we require further studies to verify the findings.

To our knowledge, AST is deposited in not only hepatocytes but also in other organs, *e.g.,* muscle, brain, and gut [48, 49]. In this case, it was reasonable that the strength of the risk of new-onset MetS with AST levels was lower than that with ALT levels. In prior studies, there were inconsistent findings in the association between increasing AST levels and MetS. Notably, some studies revealed little connection between AST levels and MetS [25-29]. For the ratio of ALT/AST, as it is a sensitive insulin resistance marker [45, 46], its attenuated association for incident MetS could be accounted for the adjustments for baseline components of MetS in the present study.

In this study, the associations of ALT and AST levels with incident MetS were particularly in women and in those without the presence of MetS features, *e.g.,* obesity and dyslipidemia. A prior meta-analysis has shown that in the presence of NAFLD, women had a greater risk of developing NASH and advanced liver fibrosis than men [50]. Since NASH were predisposed to having increased ALT and AST levels, women had a greater risk of incident MetS than men, which was consistent with our findings. In addition, since obesity and dyslipidemia may contribute to the development of NAFLD and NASH [28, 29], the presence of any of the characteristics of MetS at baseline may be a competing risk to the plasma hepatic enzymes for incident MetS and thus reduce their associations.

## 5 STUDY STRENGTHS AND LIMITATIONS

This study had some limitations. First, our cohort included only armed forces personnel, which may not be generalizable to the general population of young adults. However, in this study, the incidence of new-onset MetS (23.0%) was close to the rates reported in other studies, ranging from 21.1% to 28.9% [8-12], which suggests a high degree of comparability with broader populations. Second, information on substance use and PA levels was mainly obtained from self-reports, which might have a bias. Third, the lack of non-invasive tests, such as abdominal sonography, for the diagnosis of NAFLD is a major concern for the quality of the study. Fourth, there was no information on hemoglobin A1c and insulin which were crucial determinants to evaluate insulin resistance severity. Since our participants were young adults free of diabetes mellitus and some covariates in the adjusted models, such as TG and HDL-C, had a high correlation with the insulin resistance indices, the effect of lacking hemoglobin A1c or insulin adjustment at baseline might be minimized. Fourth, alcohol intake should be an important confounder in the association of hepatic transaminases and incident MetS, whereas there was no significant difference between those with current active alcohol intake and those with never or former alcohol intake. The possible explanation is that as alcoholic beverages were forbidden in military bases, our study participants could only consume small amounts of alcohol out of base during holidays or vacations and were unlikely to be heavy or moderate consumers. Therefore, the alcohol intake status may not significantly confound the association between hepatic transaminases and the risk of new-onset MetS.

Fifth, other causes of increased AST and ALT levels for this population, such as dehydration, rhabdomyolysis, infection, and medication, could affect the results of the study. Finally, viral hepatitis markers should be checked annually as viral hepatitis is an important cause of elevated hepatic transaminase enzymes.

## CONCLUSION

Plasma hepatic transaminase levels were positively associated with incident MetS in young adults. A modest increase in plasma ALT (≥19 U/L in men and ≥13 U/L in women) and AST levels should be focused due to an increased risk of new-onset MetS development before midlife, particularly in women and in those without any MetS feature at baseline. Accordingly, a hepatic sonography is warranted for further confirmation of the presence of NAFLD or NASH.

## Figures and Tables

**Fig. (1):**
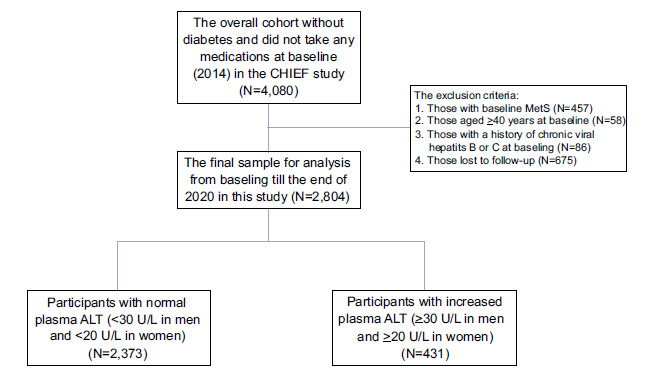
The flow diagram reveals the selection of participants in this cohort study.

**Table 1:** Characteristics of the military personnel with and without increased alanine transaminase.

-	ALT within normal limits (N =2,373)	Increased ALT (N =431)	*p*-value
Sex, %	27.94 ± 5.81	29.90 ± 5.44	<0.001
Men	-	-	-
Women	2096 (88.3)	404 (93.7)	0.001
Body mass index, kg/m^2^	277 (11.7)	27 (6.3)	-
Waist circumference, cm	23.87 ± 2.89	26.31 ± 2.77	<0.001
Systolic blood pressure, mmHg	80.36 ± 7.76	86.93 ± 7.59	<0.001
Diastolic blood pressure, mmHg	115.33 ± 12.97	118.76 ± 12.25	<0.001
Mean blood pressure, mmHg	68.86 ± 9.62	71.07 ± 9.38	<0.001
Substance use, %	99.61 ± 11.86	102.38 ± 12.48	<0.001
Alcohol drinking	-	-	-
Betel nut chewing	928 (39.1)	192 (44.5)	0.03
Cigarette smoking	207 (8.7)	65 (15.1)	<0.001
Physical activity level, %	807 (34.0)	159 (36.9)	0.24
<150 minute/week	-	-	-
150 - 299 minute/week	531 (22.4)	79 (18.3)	0.11
≥300 minute/week	894 (37.7)	180 (41.8)	-
Blood test	948 (39.9)	172 (39.9)	-
ALT, U/L	-	-	-
AST, U/L	15.55 ± 5.83	46.15 ± 22.05	<0.001
ALT/AST ratio	17.81 ± 4.96	29.67 ± 11.85	<0.001
Total cholesterol, mg/dL	0.87 ± 0.25	1.57 ± 0.42	<0.001
LDL-C, mg/dL	168.74 ± 30.74	187.52 ± 34.85	<0.001
HDL-C, mg/dL	101.01 ± 27.65	117.20 ± 30.44	<0.001
Serum triglycerides, mg/dL	50.39 ± 10.04	48.02 ± 9.52	<0.001
Fasting glucose, mg/dL	90.79 ± 48.74	128.41 ± 87.25	<0.001
Insulin resistance index	91.70 ± 9.52	93.56 ± 11.06	<0.001
TG/HDL-C	-	-	-
TyG index	1.92 ± 1.24	2.84 ± 2.24	<0.001
METS-IR	8.21 ± 0.47	8.54 ± 0.53	<0.001

**Note: ** Data are presented as mean (standard deviation) and numbers (percentage).

Definition: Increased ALT is defined as plamsa ALT >30 U/L in men and >20 U/L in women; mean blood pressure is defined as 1/3* systolic blood pressure + 2/3* diastolic blood pressure.

Increased ALT was defined as plasma ALT ≥30 U/L in men and ≥20 U/L in women.

**Abbreviations:** ALT, alanine transaminase; AST; aspartate transaminase; HDL-C, high-density lipoprotein cholesterol.

**Table 2:** Correlations of plasma hepatic transaminases with lipid profiles, fasting glucose and insulin resistance indices.

-	Plasma ALT (each 10 U/L higher)			Plasma AST (each 10 U/L higher)			ALT/AST (each 1 unit higher)		
-	r	standardized β	*p*-value	r	standardized β	*p*-value	r	standardized β	*p*-value
TC	0.413	0.169	<0.001	0.312	0.122	<0.001	0.466	0.167	<0.001
LDL-C	0.405	0.144	<0.001	0.295	0.058	0.003	0.473	0.187	<0.001
HDL-C	0.382	-0.018	0.32	0.302	0.089	<0.001	0.451	-0.108	<0.001
TG	0.421	0.188	<0.001	0.315	0.131	<0.001	0.471	0.181	<0.001
FPG	0.382	0.003	0.85	0.292	-0.031	0.09	0.441	0.035	0.04
TG/HDL-C	0.416	0.177	<0.001	0.304	0.097	<0.001	0.475	0.191	<0.001
TyG index	0.415	0.180	<0.001	0.301	0.088	<0.001	0.482	0.218	<0.001
METS-IR	0.403	0.150	<0.001	0.291	0.019	0.38	0.481	0.229	<0.001

**Note: ** Multivariable linear regression analysis with adjustments for age, sex, alcohol drinking, betel nut chewing, cigarette smoking, physical activity and body mass index.

Pearson correlation coefficient was denoted as “r”.

**Abbreviations:** ALT, alanine transaminase; AST, aspartate transaminase; TC, total cholesterol; LDL-C, low-density lipoprotein cholesterol; HDL-C, high-density lipoprotein cholesterol; TG, triglycerides; FPG, fasting glucose; TyG index, triglyceride glucose index; METS-IR, metabolic score for insulin resistance.

**Table 3:** Multivariable cox regression analysis for incidence of metabolic syndrome with plasma hepatic transaminases.

ALT (U/L)	Crude Model			-	Model 1	-	-	Model 2	-
-	HR	95% CI	*p*	HR	95% CI	*p*	HR	95% CI	*p*
ALT (each 10 U/L higher)	1.18	1.16 - 1.21	<0.001	1.09	1.06 - 1.31	<0.001	1.05	1.01 - 1.09	0.01
Sex-specific Q1 (ref)	1.00	-	-	1.00	-	-	1.00	-	-
Sex-specific Q2	1.52	1.12 - 2.05	0.007	1.06	0.78 - 1.43	0.72	1.07	0.79 - 1.45	0.67
Sex-specific Q3	2.70	2.06 - 3.54	<0.001	1.50	1.14 - 1.99	0.004	1.33	1.00 - 1.76	0.04
Sex-specific Q4	4.06	3.13 - 5.27	<0.001	1.69	1.28 - 2.23	<0.001	1.42	1.07 - 1.88	0.01
AST (each 10 U/L higher)	1.35	1.28 - 1.43	<0.001	1.16	1.08 - 1.24	<0.001	1.11	1.04 - 1.19	0.003
Sex-specific Q1 (ref)	1.00	-	-	1.00	-	-	1.00	-	-
Sex-specific Q2	1.35	1.04 - 1.77	0.02	1.01	0.77 - 1.32	0.94	1.04	0.79 - 1.37	0.76
Sex-specific Q3	1.36	1.06 - 1.73	0.01	0.98	0.77 - 1.26	0.87	1.06	0.82 - 1.36	0.67
Sex-specific Q4	2.40	1.92 - 3.01	<0.001	1.34	1.06 - 1.69	0.01	1.29	1.01 - 1.65	0.03
ALT/AST (each 1 unit higher)	2.28	2.42 - 3.29	<0.001	1.46	1.21 - 1.77	<0.001	1.12	0.92 - 1.37	0.26
Sex-specific Q1 (ref)	1.00	-	-	1.00	-	-	1.00	-	-
Sex-specific Q2	1.67	1.25 - 2.23	0.001	1.24	0.93 - 1.66	0.15	1.21	0.91 - 1.63	0.19
Sex-specific Q3	2.76	2.10 - 3.63	<0.001	1.59	1.20 - 2.10	0.001	1.31	0.99 - 1.74	0.06
Sex-specific Q4	3.84	2.95 - 4.99	<0.001	1.72	1.30 - 2.26	<0.001	1.31	0.99 - 1.73	0.06

**Note: ** Data are presented as hazard ratio (HR) and 95% confidence interval (CI) using multivariable Cox regression analysis for Model 1: age, sex, alcohol drinking, betel nut chewing, cigarette smoking, physical activity and body mass index adjustments;

Model 2: Model 2 + waist circumference, mean blood pressure (1/3* systolic blood pressure + 2/3* diastolic blood pressure), high-density lipoprotein, serum triglycerides and fasting glucose adjustments.

Definitions: ALT Q1 was defined as <13 U/L in men and <8 U/L in women; ALT Q2 was defined as 13-16 U/L in men and 8-10 U/L in women; ALT Q3 was defined as 17 -24 U/L in men and 11-13 U/L in women; ALT Q4 was defined as ≥25 U/L in men and ≥14 U/L in women. AST Q1 was defined as <16 U/L in men and <13 U/L in women; AST Q2 was defined as 16-17 U/L in men and 13-14 U/L in women; AST Q3 was defined as 18 -21 U/L in men and 15-17.4 U/L in women; AST Q4 was defined as ≥22 U/L in men and ≥17.5 U/L in women. ALT/AST Q1 was defined as <0.75 in men and <0.59 in women; ALT/AST Q2 was defined as 0.75-0.93 in men and 0.59-0.69 in women; ALT/AST Q3 was defined as 0.94-1.20 in men and 0.70-0.84 in women; ALT/AST Q4 was defined as ≥1.21 in men and ≥0.85 in women.

**Abbreviations:** ALT, alanine transaminase; AST; aspartate transaminase; ref, reference.

**Table 4:** Associations of plasma hepatic transaminases and its ratio with incident metabolic syndrome in young adults in subgroup analyses.

-	-	Plasma ALT (each 10 U/L higher)				Plasma AST (each 10 U/L higher)				ALT/AST ratio (each 1 unit higher)			
Baseline	N	HR	95% CI	*p*	*p*-value for interaction	HR	95% CI	*p*	*p*-value for interaction	HR	95% CI	*p*	*p*-value for interaction
Sex	-	-	-	-	-	-	-	-	-	-	-	-	-
Men	2,500	1.05	1.01 - 1.09	0.02	0.06	1.10	1.02 - 1.18	0.01	0.001	1.10	0.90 - 1.35	0.35	0.57
Women	304	1.75	1.07 - 2.87	0.02	-	2.14	1.35 - 3.41	0.001	-	2.20	0.46 -10.60	0.32	-
BMI	-	-	-	-	-	-	-	-	-	-	-	-	-
≥25 kg/m^2^	1,080	1.04	0.99 - 1.08	0.12	0.02	1.07	0.99 - 1.16	0.10	0.001	1.07	0.84 - 1.35	0.59	0.20
<25 kg/m^2^	1,724	1.08	1.00 - 1.17	0.04	-	1.33	1.14 - 1.56	<0.001	-	1.10	0.74 - 1.61	0.64	-
*Waist circumference	-	-	-	-	-	-	-	-	-	-	-	-	-
With central obesity	488	1.02	0.95 - 1.09	0.63	0.04	1.05	0.92 - 1.19	0.50	0.07	1.05	0.76 - 1.47	0.75	0.14
Without central obesity	2,316	1.07	1.02 - 1.11	0.003	-	1.14	1.06 - 1.23	0.001	-	1.17	0.91 - 1.51	0.23	-
Blood pressure	-	-	-	-	-	-	-	-	-	-	-	-	-
≥130/85 mmHg	437	1.01	0.94 - 1.09	0.78	0.22	1.02	0.91 - 1.15	0.75	0.08	1.03	0.67 - 1.60	0.89	0.56
<130/85 mmHg	2,367	1.07	1.02 - 1.12	0.005	-	1.18	1.08 - 1.30	<0.001	-	1.14	0.91 - 1.43	0.26	-
**Low HDL-C	-	-	-	-	-	-	-	-	-	-	-	-	-
Presence	433	1.00	0.93 - 1.07	0.99	0.06	1.08	0.94 - 1.24	0.27	0.35	0.84	0.55 - 1.27	0.41	0.23
Absence	2,457	1.08	1.03 - 1.14	0.001	-	1.12	1.04 - 1.22	0.004	-	1.20	0.96 - 1.52	0.11	-
Serum triglycerides	-	-	-	-	-	-	-	-	-	-	-	-	-
≥150 mg/dL	337	1.02	0.95 - 1.11	0.55	0.07	1.06	0.93 - 1.20	0.39	0.07	1..13	0.77 - 1.66	0.54	0.43
<150 mg/dL	2,553	1.06	1.02 - 1.11	0.006	-	1.17	1.08 - 1.27	<0.001	-	1.02	0.81 - 1.30	0.84	-
Fasting glucose	-	-	-	-	-	-	-	-	-	-	-	-	-
≥100 mg/dL	414	1.06	0.96 - 1.18	0.27	0.97	1.18	0.97 - 1.44	0.10	0.35	1.18	0.73 - 1.91	0.49	0.55
<100 mg/dL	2,390	1.04	1.00 - 1.09	0.04	-	1.09	1.01 - 1.18	0.02	-	1.10	0.88 - 1.38	0.39	-
Alcohol consumption	-	-	-	-	-	-	-	-	-	-	-	-	-
Current active	1,120	1.09	1.04 - 1.14	<0.001	0.62	1.13	1.04 - 1.24	0.006	0.38	1.39	1.07 - 1.82	0.01	0.63
Former/never	1,684	1.11	1.05 - 1.17	<0.001	-	1.21	1.09 - 1.34	<0.001	-	1.60	1.22 - 2.08	0.001	-
Tobacco smoking	-	-	-	-	-	-	-	-	-	-	-	-	-
Current active	966	1.06	1.01 - 1.12	0.02	0.08	1.10	0.99 - 1.22	0.08	0.07	1.29	0.96 - 1.73	0.09	0.11
Former/never	1,838	1.13	1.08 - 1.18	<0.001	-	1.23	1.12 - 1.34	<0.001	-	1.66	1.29 - 2.12	<0.001	-

**Note: ** Data are presented as hazard ratio (HR) and 95% confidence interval (CI) using multivariable Cox regression analysis for age, sex, alcohol drinking, betel nut chewing, cigarette smoking, physical activity levels and body mass index.

*Central obesity is defined as waist circumference ≥90 cm for men and ≥80 cm for women; **Low HDL-C <40 mg/dL in men and <50 mg/dL in women.

**Abbreviations:** ALT, alanine aminotransferase; AST; aspartate aminotransferase; HDL-C, high-density lipoprotein cholesterol.

## Data Availability

The datasets generated and/or analyzed during the current study are not publicly available due to materials obtained from the military in Taiwan, which were confidential, but are available from the corresponding author on reasonable request.

## LIST OF ABBREVIATIONS

ALT: Alanine Transaminase
AST: Aspartate Transaminase
AUC: Area Under Curve
BMI: Body Mass Index
BP: Blood Pressure
CHIEF: Cardiorespiratory Fitness and Health in Eastern Armed Forces
CI: Confidence Intervals
eGFR: Estimated Glomerular Filtration Rate
HDL: C High-Density Lipoprotein Cholesterol
HOMA: Homeostatic Model Assessment
HR: Hazard Ratio
IDF: International Diabetes Federation
IR: Insulin Resistance
LDL: C Low-Density Lipoprotein Cholesterol
MetS: Metabolic Syndrome
METS: IR Metabolic Score for Insulin Resistance
NAFLD: Non-Alcoholic Fatty Liver Disease
NASH: Non-Alcoholic Steatohepatitis
ROC: Receiver Operating Characteristic
SD: Standard Deviation
TG: Triglyceride
TyG: Triglyceride Glucose

## STANDARDS OF REPORTING

STROBE guidelines were followed.
