# Supplementary material for: Plasma Hepatic Transaminases and Incidence of Metabolic Syndrome Before Midlife in Military Adults: A CHIEF Cohort Study
Source: Endocr Metab Immune Disord Drug Targets. 2025 Jan 9;25(7):582–92. doi: 10.2174/0118715303326392241022050205 (PMC12481545; doi:10.2174/0118715303326392241022050205)
Supplement: Supplementary file 1 [file EMIDDT-25-7-582_SD1.pdf]

Supplementary Material

Plasma Hepatic Transaminases and Incidence of Metabolic Syndrome Before Midlife in Military Adults: A CHIEF Cohort Study

Fang-Chen Liu[1, 2], Kai-Wen Chen [1], Kun-Zhe Tsai[1, 3], Chen-Chih Chu[2], Yen-Chen Lin[4], Yun-Chen Chang[5, 6], Gen-Min Lin[1, 2*]

[1.]Department of Medicine, Hualien Armed Forces General Hospital, Hualien City, Taiwan; [2.]Department of Medicine, Tri-Service General Hospital, National Defense Medical Center, Taipei, Taiwan [3.]Department of Stomatology of Periodontology, Mackay Memorial Hospital, Taipei, Taiwan; [4.]Department of Medicine, Linkou Chang Gung Memorial Hospital, Taoyuan, Taiwan; [5.]School of Nursing and Graduate Institute of Nursing, China Medical University, Taichung, Taiwan; [6.]Department of Nursing, China Medical University Hospital, Taichung, Taiwan.

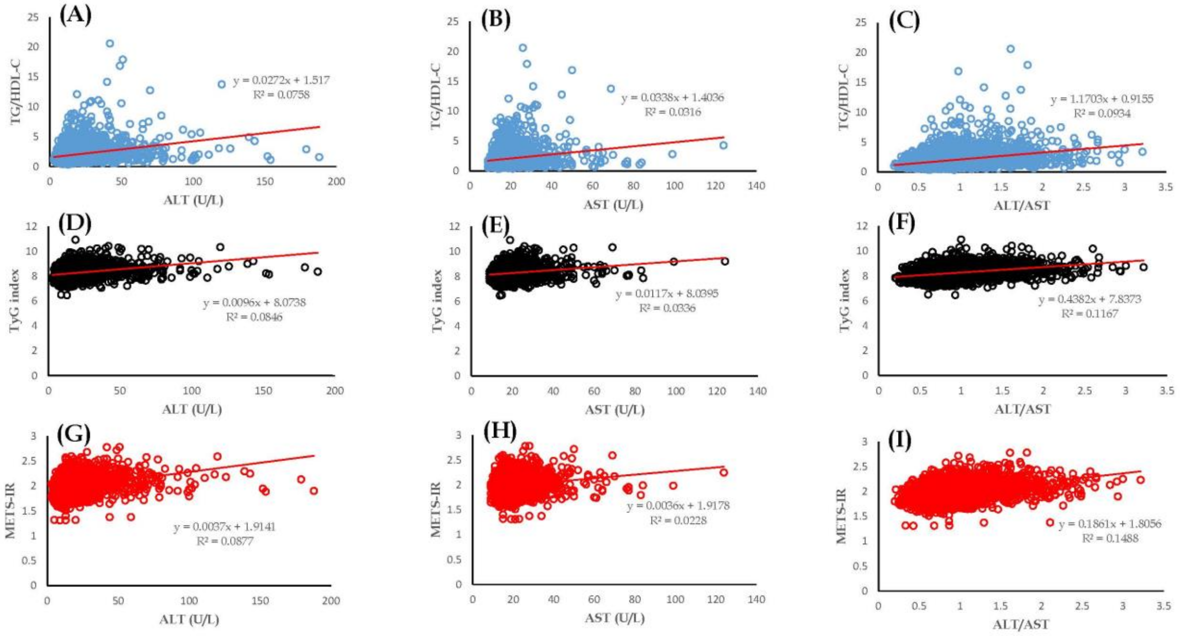

Fig. (S1). Graphical representation of TG/HDL-C, Tyg-index, MIETS-IR.

Table S1. Multivariable Cox Regression Analysis for Incidence of Metabolic Syndrome with Plasma Alanine Transaminase.

ALT (U/L)					Crude Model			Model 1			Model 2	
	N	Events	Power	HR	95% CI	*p*	HR	95% CI	*p*	HR	95% CI	*p*
Normal ALT (ref)	2373	469		1.00			1.00			1.00		
Increased ALT	431	175	100%	2.30	1.94 – 2.73	<0.001	1.21	1.01 – 1.45	0.03	1.00	0.84 – 1.21	0.96
Normal ALT (ref)	2205	416										
*Increased ALT	599	228	100%	2.25	1.92 – 2.64	<0.001	1.32	1.12 – 1.56	0.001	1.10	0.93 – 1.31	0.28
Normal ALT (ref)	1629	233										
**Increased ALT	1175	411	100%	2.68	2.29 – 3.15	<0.001	1.51	1.27 – 1.79	0.001	1.28	1.08 – 1.53	0.005

Data are present as hazard ratio (HR) and 95% confidence interval (CI) using multivariable Cox regression analysis with adjustments for age, sex, alcohol drinking, betel nut chewing, cigarette smoking, physical activity and body mass index in Model 1; and additionally for waist circumference, mean blood pressure (1/3* systolic blood pressure + 2/3* diastolic blood pressure), high-density lipoprotein, serum triglycerides and fasting glucose in Model 2.

*Increased ALT is defined as plasma ALT >27 U/L in men and >15 U/L in women

**Increased ALT is defined as plasma ALT >19 U/L in men and >13 U/L in women according to the ROC analysis results in supplemental Table 2.

Abbreviations: ALT, alanine transaminase; ref, reference

Table S2. Correlations Between Lipid Profiles, Fasting Glucose and Insulin Resistance Indices.

	TC	LDL-C	HDL-C	TG	FPG	TG/HDL-C	TyG index	METS-IR
TC	1.000	0.893	0.147	0.366	0.084	0.274	0.404	0.213
LDL-C	0.893	1.000	-0.167	0.227	0.067	0.216	0.322	0.321
HDL-C	0.147	-0.167	1.000	-0.261	-0.063	-0.464	-0.300	-0.733
TG	0.366	0.227	-0.261	1.000	0.097	0.957	0.899	0.755
FPG	0.084	0.067	-0.063	0.097	1.000	0.098	0.280	0.117
TG/HDL-C	0.274	0.216	-0.464	0.957	0.098	1.000	0.852	0.847
TyG index	0.404	0.322	-0.300	0.899	0.280	0.852	1.000	0.815
METS-IR	0.213	0.321	-0.733	0.755	0.117	0.847	0.815	1.000

Data are presented as Pearson correlation coefficient (r).

Abbreviations: TC, total cholesterol; LDL-C, low-density lipoprotein cholesterol; HDL-C, high-density lipoprotein cholesterol; TG, triglycerides; FPG, fasting glucose; TyG index, triglyceride glucose index; METS-IR, metabolic score for insulin resistance.

Table S3. Sex-Specific Receiver Operating Characteristics Analysis of Hepatic Transaminases for Incident Metabolic Syndrome.

	Sensitivity	Specificity	Cut-off point	AUC	95% CI	*p*-value
Men						
ALT	0.644	0.628	18.5	0.674	0.651 – 0.697	<0.001
AST	0.415	0.751	21.5	0.615	0.590 – 0.640	<0.001
ALT/AST	0.679	0.583	0.945	0.658	0.634 – 0.682	<0.001
Women						
ALT	0.692	0.707	12.5	0.729	0.625 – 0.834	<0.001
AST	0.346	0.887	19.5	0.615	0.488 – 0.742	0.052
ALT/AST	0.731	0.707	0.787	0.718	0.614 – 0.822	<0.001

Receiver Operating Characteristic (ROC) analysis with age, sex, alcohol drinking, betel nut chewing, cigarette smoking, physical activity levels and body mass index adjustments

Abbreviations: ALT, alanine transaminase; AST; aspartate transaminase; AUC, area under curve; CI, confidence interval
